# Polar or Apolar—The Role of Polarity for Urea-Induced Protein Denaturation

**DOI:** 10.1371/journal.pcbi.1000221

**Published:** 2008-11-14

**Authors:** Martin C. Stumpe, Helmut Grubmüller

**Affiliations:** Department of Theoretical and Computational Biophysics, Max-Planck-Institute for Biophysical Chemistry, Göttingen, Germany; Stanford University, United States of America

## Abstract

Urea-induced protein denaturation is widely used to study protein folding and stability; however, the molecular mechanism and driving forces of this process are not yet fully understood. In particular, it is unclear whether either hydrophobic or polar interactions between urea molecules and residues at the protein surface drive denaturation. To address this question, here, many molecular dynamics simulations totalling ca. 7 µs of the CI2 protein in aqueous solution served to perform a computational thought experiment, in which we varied the polarity of urea. For apolar driving forces, hypopolar urea should show increased denaturation power; for polar driving forces, hyperpolar urea should be the stronger denaturant. Indeed, protein unfolding was observed in all simulations with decreased urea polarity. Hyperpolar urea, in contrast, turned out to stabilize the native state. Moreover, the differential interaction preferences between urea and the 20 amino acids turned out to be enhanced for hypopolar urea and suppressed (or even inverted) for hyperpolar urea. These results strongly suggest that apolar urea–protein interactions, and not polar interactions, are the dominant driving force for denaturation. Further, the observed interactions provide a detailed picture of the underlying molecular driving forces. Our simulations finally allowed characterization of CI2 unfolding pathways. Unfolding proceeds sequentially with alternating loss of secondary or tertiary structure. After the transition state, unfolding pathways show large structural heterogeneity.

## Author Summary

To perform their physiological function, proteins have to fold into their characteristic three-dimensional structure. While the folded state is stable under physiological conditions, changes in the solvent can destabilize the folded state and even induce denaturation. One of the most commonly used denaturants is urea. Despite its widespread use to study protein folding and stability, however, the molecular mechanism and particularly the driving forces of urea-induced protein denaturation are not yet understood. Two mechanisms have been suggested, according to which denaturation is driven either by polar interactions via hydrogen bonds or by hydrophobic interactions with apolar amino acids. By systematically varying urea polarity and quantifying the interactions of the solvent molecules with all amino acids of the protein, the present simulation study reveals that it is mainly the apolar interactions that drive denaturation. Our results suggest a coherent microscopic picture for urea-induced denaturation and bear more general implications for protein stability in other environments, e.g., in chaperone-assisted folding.

## Introduction

Protein denaturation by osmolytes such as urea or guanidinium is widely used to study protein folding and stability. The underlying mechanism, however, is not yet fully understood on the molecular level. Despite the large number of theoretical and experimental studies carried out in the past decades to shed light on the molecular details of this process, no clear picture has emerged yet. On the one hand, the microsecond to millisecond timescales at which individual folding and unfolding events occur, as well as the need for synchronization in ensemble measurements, and the structural heterogeneity of unfolding pathways renders it difficult to gain atomistic insight from experiments. On the other hand, for computer simulations, folding/unfolding processes are typically too slow or too rare to be accessible.

Two basic model classes have guided the study of the driving forces of urea-induced protein denaturation, and still set the framework for ongoing discussions. According to the first model, urea induces changes in the water structure, which in turn weaken the hydrophobic effect and thus cause protein denaturation [1]–[3]. In this model of *indirect* interactions, two alternative views have been put forward in which urea is regarded either to break [1],[2], or to enhance [3] water structure. The second model, in contrast, attributes the denaturing effect of urea to *direct* interactions between urea and the protein [4]–[6]. Also this model comprises different aspects: either the interaction of urea with polar residues or the peptide backbone, mainly via hydrogen bonding [5]—or hydrophobic interaction with apolar residues [4].

All of these possibilities, and various combinations thereof, have been suggested as the primary driving force of denaturation, and are still controversially discussed. Whereas some studies have provided support for the primacy of indirect effects [7]–[12], this concept has been challenged by many authors [6],[13],[14], and many recent studies provide increasing evidence for direct interactions as the primary driving force for denaturation [14]–[25]. Within this framework, however, it is controversially discussed whether either polar [11],[12],[17],[22],[23],[25] or apolar [4], [15], [16], [18]–[21],[24],[26] interactions between urea and the protein dominate.

Here we address this question by studying the relevance of direct polar and apolar contacts with all-atom molecular dynamics (MD) simulations. We have chosen the chymotrypsin inhibitor 2 (CI2) protein as an example, the folding kinetics and thermodynamics of which have been extensively studied experimentally [27]. We consider the CI2 in water as well as in aqueous urea solution, and perform a thought experiment (“Gedankenexperiment”), in which urea polarity is varied by scaling its partial atomic charges. The rational of this computer experiment is as follows. If polar contacts such as hydrogen bonds between urea and the protein constituted the determinant interaction for denaturation, one would expect hyperpolar urea to be an even stronger denaturant than real urea. If, in contrast, apolar contacts played the major role for denaturation, one would expect hypopolar urea to be the stronger denaturant. Therefore, by monitoring the respective denaturation strengths in the simulations, we will be able to decide which of the two interaction types drives urea-induced unfolding.

## Methods

### Simulation Setup

All simulations were performed using the Gromacs [28]–[30] program suite, versions 3.2.1 and 3.3, with the OPLS-all-atom force-field [31],[32]. The TIP4P water model [33] was used, and the urea force field was adopted from Smith et al. [34], which is a refined version of the original OPLS parametrization by Duffy et al. [18]. A cutoff of 1.0 nm was used for short-range Coulomb as well as Lennard-Jones interactions. Particle Mesh Ewald summation (PME) [35],[36] was used to calculate the long-range electrostatic interactions with a grid-spacing of 0.12 nm and an interpolation order of 4. All simulations were performed in the *N_p_T*-ensemble using Berendsen-type temperature-coupling [37] with a coupling coefficient of *τ_T_* = 0.1 ps and Berendsen-type pressure-coupling [37] at 1 bar with a coupling coefficient of *τ_p_* = 1 ps. To allow comparison with the simulations reported in [11], the simulation temperature was set to 333 K (except for one simulation at 300 K), and the same CI2 double mutant (E33A, E34A) was used. An integration timestep of 2 fs was used together with the LINCS constraint solver [38] for all covalent bonds.

The structure of the CI2 protein was taken from the Protein Data Bank [39], PDB-code 1YPC [40]. Unresolved side chain atoms for residue MET40 (residue number 59 in the pdb file) were added using the program WHAT IF [41]. The box-size was chosen such that a minimum distance of 1.5 nm between protein atoms and the box was kept. For the solvation of the protein, pre-equilibrated structures of water and 8 M urea were used (taken from [42]). Sodium and chloride ions were added to yield a 150 mM ion concentration and mimic physiological conditions. Prior to each simulation, a 200 step steepest descent energy minimization and a 500 ps equilibration run with position restraints on the protein heavy atoms were carried out.

To avoid over-interpretation of possibly anecdotal events, multiple simulation runs were carried out for each parameter set (Table 1). Two simulations of CI2 in water, three simulations with regular urea charges, two simulations with 25% urea charges, five simulations with 50% urea charges, four simulations with 75% urea charges, two simulations with 150% urea charges and two simulations with 200% urea charges were performed, each at 333 K. In addition, one simulation in water at 300 K was performed to define native contacts and native secondary structure (see below). The total simulation time of all simulations was ca. 7 µs.

**Table 1:** Solvent, partial charge scaling, and length of all 22 simulation runs discussed in the text.

Label	Solvent	Scaling Factor for Urea Partial Charges	Simulation Time [ns]
W^300 *K*^	Water (300 K)	–	100 ns
W^1^	Water	–	285 ns
W^2^	Water	–	500 ns
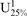	8 M Urea	25%	378 ns
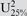	8 M Urea	25%	300 ns
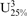	8 M Urea	25%	435 ns
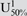	8 M Urea	50%	176 ns
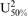	8 M Urea	50%	357 ns
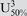	8 M Urea	50%	395 ns
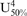	8 M Urea	50%	296 ns
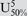	8 M Urea	50%	289 ns
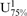	8 M Urea	75%	332 ns
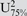	8 M Urea	75%	225 ns
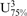	8 M Urea	75%	250 ns
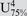	8 M Urea	75%	250 ns
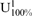	8 M Urea	100%	402 ns
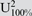	8 M Urea	100%	285 ns
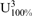	8 M Urea	100%	522 ns
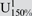	8 M Urea	150%	461 ns
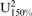	8 M Urea	150%	500 ns
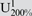	8 M Urea	200%	277 ns
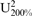	8 M Urea	200%	234 ns
Total simulation time:			7249 ns

We note that a computational thought experiment not dissimilar to the one performed here was conducted by Sorin et al. [43], who investigated the relationship between solvent and protein structure in a “hydrophobic titration” experiment employing different TIP3P variants.

### Analysis

Solvent accessible hydrophobic surface areas (SAS) were calculated using the double cubic lattice method [44] with a 0.14 nm probe radius. Native contacts and native secondary structure were defined using the simulation at 300 K in water (W^300 *K*^), rather than the crystal structure. This approach has the advantage that fluctuations of the native state were captured which allowed a more direct comparison with the unfolding simulations. Residues were defined to be in contact if the distance between the closest atom pair was not larger than 0.4 nm. Contacts were defined as native if they were present during more than 50% of the time in simulation W^300*K*^. Contacts between neighboring residues were not considered for the calculation of the native contact fraction.

Secondary structure was classified using DSSP [45]. The native secondary structure was defined as the most frequently occurring structure type for each residue seen in simulation W^300*K*^, which was similar to that of the crystal structure. Helix, *β*-sheet, and turn-elements were considered to calculate the fraction of native secondary structure content.

### Contact Coefficient

To quantify the frequency of interactions between urea and the amino acids, we used the contact coefficient *C_UW_* [46] for a particular amino acid X,
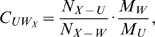
(1)where *N_X–U_* and *N_X–W_* are the numbers of atomic contacts of amino acid X with urea and water molecules, respectively. Atoms were defined to be in contact if close than 0.35 nm. *C_UW_* is normalized using the total numbers of urea atoms (*M_U_*) and water atoms (*M_W_*). Accordingly, a residue with a contact coefficient of *C_UW_* = 1.0 has no interaction preference for either urea or water. Values above 1.0 indicate preferential interaction with urea, values below 1.0 indicate preferential interaction with water.

## Results

### The Native State in Water/Urea

**Figure 1:**
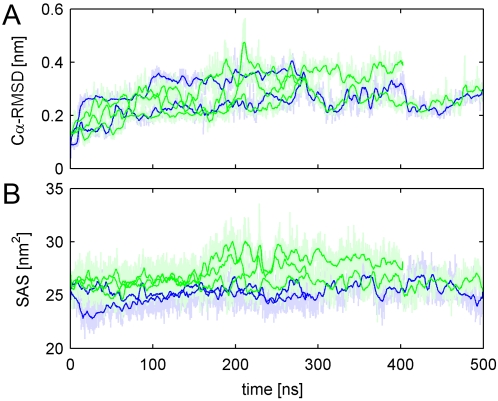
CI2 in native conformation. (A) C*_α_*-RMSD. (B) SAS for the two simulations in water (blue) and the 3 simulations in aqueous urea with regular charges (green). The solid bold lines show traces smoothed by a running average over 500 ps; dim lines show raw data.

As a reference, we first analyzed the dynamics and stability of the folded CI2 protein as well as its protein-solvent interactions both in water and in 8 M aqueous urea solution. Figure 1 shows the C*_α_* root-mean-square-deviation (RMSD, panel A) and the solvent accessible hydrophobic surface area (SAS, panel B) for the simulations in water (W^1,2^, blue) and in 8 M urea solution (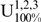, green). As can be seen, the C*_α_*-RMSD of the protein in both solvents shows similar fluctuations with an average value of 0.3 nm, and no significant differences between both solvents are seen. In particular, no unfolding is observed, which is expected from the measured millisecond time scale for CI2 denaturation [47].

In contrast, and perhaps unexpectedly, the average SAS in aqueous urea is 2–3 nm^2^ larger than in water. As can be seen in Figure 1B, this difference is significantly larger than the SAS fluctuations of single trajectories. Closer inspection reveals that this difference results mainly from few specific residues whose side chains are more solvent-exposed in aqueous urea than in water. In particular, MET1, LEU32, ILE44 and PHE50 contribute dominantly to this difference (0.22 nm^2^, 0.17 nm^2^, 0.30 nm^2^ and 0.29 nm^2^, respectively). With only a few exceptions (e.g., ARG43), however, also the side chains of almost all other residues are slightly more exposed in aqueous urea solution than in water. Because these amino acids are among those which were found to have particularly strong contact preferences for urea (see [46]), we expect that the increased exposure of these side chains is caused by favorable interactions with urea molecules.

**Figure 2:**
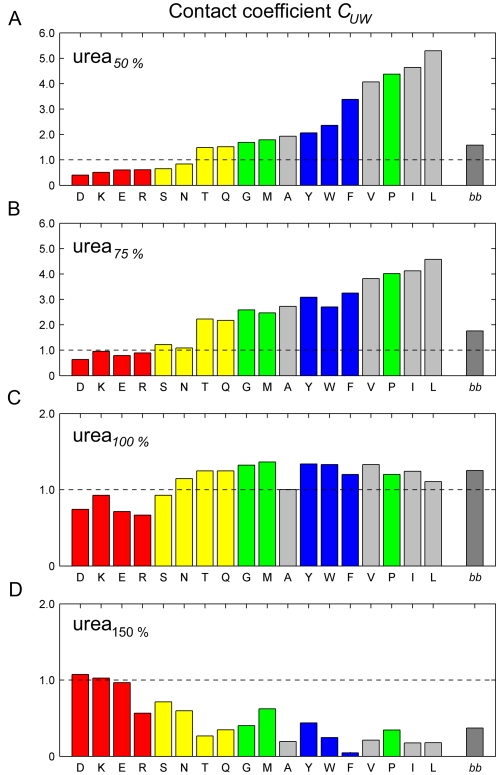
Interaction coefficient *C_UW_* for all amino acids types in the CI2 protein, as well as the backbone average (“*bb*”). The four panels show *C_UW_* for the different urea partial charge scalings (A: 50%, B: 75%, C: 100%, D: 150%). The color characterizes the amino acids. Red: charged, yellow: polar, gray: aliphatic, blue: aromatic, green: apolar. For better comparability, all *C_UW_* are sorted according to *C_UW_* in urea_50%_.

To check whether this trend holds not only for tripeptides [46], but also for the whole protein, we quantified these interactions using the contact coefficient C*_UW_*. Figure 2C shows the *C_UW_* values for each amino acid type in the CI2, averaged over time and over the three simulations in aqueous urea solution (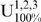). Indeed, the obtained contact coefficients are largely similar to those calculated for the individual amino acids in tripeptides [46]. In particular, apolar and aromatic amino acids, as well as the backbone, have pronounced contact preferences for urea, whereas charged amino acids have preferences for water contact. This finding confirms that polarity/apolarity is clearly a determining factor for the specific interactions of urea with the CI2 protein residues, and provides further motivation for our approach to investigate protein stability in solutions of urea with modified polarity.

We note that the remaining differences between the contact coefficients of tripeptides versus those observed here for CI2—quantified by a correlation coefficient of *r* ^2^ = 0.69—suggests that effects from sequence and structure of the folded CI2 protein account for ca. 30% of the contact preferences.

### Protein Stability in Hypo- and Hyperpolar Urea

To investigate the denaturation strengths of hyper- or hypopolar urea, the partial charges of urea were scaled to values of 25%, 50%, 75%, 150%, and 200%. For each of these modified degrees of polarity, the CI2 protein was simulated in aqueous solution. Urea with partial charge scaling of *x*% will be denoted as “urea*_x_*_%_”.

Since it is a priori not clear that upscaling or downscaling urea partial charges does in fact enhance polar or apolar, respectively, interactions with the protein, we investigated the contact coefficients of each amino acid type in the CI2 for hypo- and hyperpolar urea. As can be seen in Figure 2A and 2B, hypopolar urea indeed shows less interactions with charged and polar amino acids, and enhanced interactions with less polar residues. Hyperpolar urea_150%_, in contrast, exhibits fewer interactions with those amino acids preferentially interacting with “regular” urea_100%_ (Figure 2D). Interactions with charged residues are even preferred by urea_150%_ over interactions with less polar residues. In summary, lowering the polarity of urea enhances its interaction preferences: less preferred interactions become even less frequent, and preferred interactions become even more frequent. An exception is ARG, which does not show enhanced interactions for urea_150%_. We attribute this effect to the fact that ARG contains large polar as well as apolar parts.

**Figure 3:**
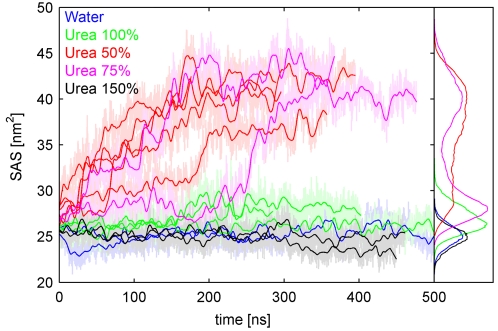
Solvent accessible surface area of the protein in all simulations. Blue: water, orange: urea_50%_, magenta: urea_75%_, green: urea_100%_, black: urea_150%_. The lines show traces smoothed by a running average over 500 ps. The histogram in the right panel shows the frequency of the respective SAS.

Having shown that upscaling or downscaling urea partial charges has the desired effect on the interaction strengths between urea and the different amino acids, we can now turn our attention to the influence on protein stability. Accordingly, we monitored the SAS for the different urea partial charge scalings (Figure 3). As can be seen, for hypopolar urea, the protein unfolds in all nine simulations (urea_75%_ and urea_50%_, magenta and orange lines, respectively). In contrast, for hyperpolar urea_150%_, the SAS remains close to the native value and the protein remains stable in all simulations (black lines). In fact, the SAS is even smaller for hyperpolar urea than for regular urea, which suggests that hyperpolar urea compacts the folded state. Furthermore, this result suggests that urea_150%_ would actually be a weaker denaturant than urea_100%_.

In summary, enhanced apolar interactions between urea and the protein destabilize the native state and induce unfolding of the CI2. Strengthening *apolar* interactions yields a *stronger* denaturant, while strengthening *polar* interactions yields a *weaker* denaturant.

We have also performed simulations with “extreme” urea_25%_ and urea_200%_. However, these simulations exhibit artifacts which render them irrelevant for the present purpose and are therefore not shown in Figure 3. For partial charges scaled down to 25%, on the one hand, urea shows a strong tendency to self-aggregate to a hydrophobic layer in the periodic simulation box, which does not any more interact with the protein. Urea_200%_, on the other hand, induces a glass transition in the solvent, with drastically reduced urea diffusion coefficients (from ≈2.2·10^−5^ cm^2^/s to <0.001·10^−5^ cm^2^/s). As a result of the vanishing mobility, the urea molecules do not interact with the protein either. Similar underestimations of the diffusion coefficients in common force-fields has previously been observed for high ion concentrations [48].

We note that these two side-effects, urea aggregation and reduced diffusion coefficients, were also observed for the simulations with urea_50%_ and urea_150%_, respectively, albeit to a (much) lesser extent. Care has to be taken, therefore, that these side-effects do not affect our main conclusions. In particular, one might argue that protein unfolding in urea_50%_ is not necessarily a direct consequence of reduced urea polarity. Rather, it might be caused by inhomogeneities of urea concentration. However, since the observed unfolding events are quite similar to those observed for urea_75%_, where no significant aggregation is seen, we do not expect locally enhanced urea concentration to play a significant role.

For the simulations with hyperpolar urea_150%_, one might object that not enhanced protein stability, but reduced urea diffusion coefficient for urea_150%_ (from ≈2.2·10^−5^ cm^2^/s to ≈0.1·10^−5^ cm^2^/s) is the reason that no unfolding is observed. To address this concern, two effects of this reduced urea mobility need to be considered. First, the reduced mobility of urea molecules implies much slower thermodynamic equilibration. And therefore, the thermodynamic equilibrium distribution at the protein surface might not be reached within the available simulation time. However, the diffusion time for a urea molecule to cross the whole box length is well within the simulations time (≈75 ns for urea_150%_), such that this effect can be excluded.

Second, the reduced mobility of urea molecules might slow down conformational changes of the protein due to higher solvent viscosity. Note, however, that conformational changes *are* seen on the simulation timescale, which lead to the observed compaction. Furthermore, as can be seen from the fast 10 ns SAS jumps in urea_75%_ and urea_50%_, even a 20-fold enhanced viscosity is unlikely to prevent motions on a 500 ns timescale. This observation, together with the fact that other proteins, e.g. the Cold Shock protein, are observed to undergo large conformational changes in hyperpolar urea_150%_ (data not shown) strongly suggests that the increased solvent viscosity does not compromise our interpretation.

The extent of both side-effects, self-diffusion slowdown and urea aggregation, is shown in the Supporting Information (Text S1).

### Unfolding Pathways in Hypopolar Urea

**Figure 4:**
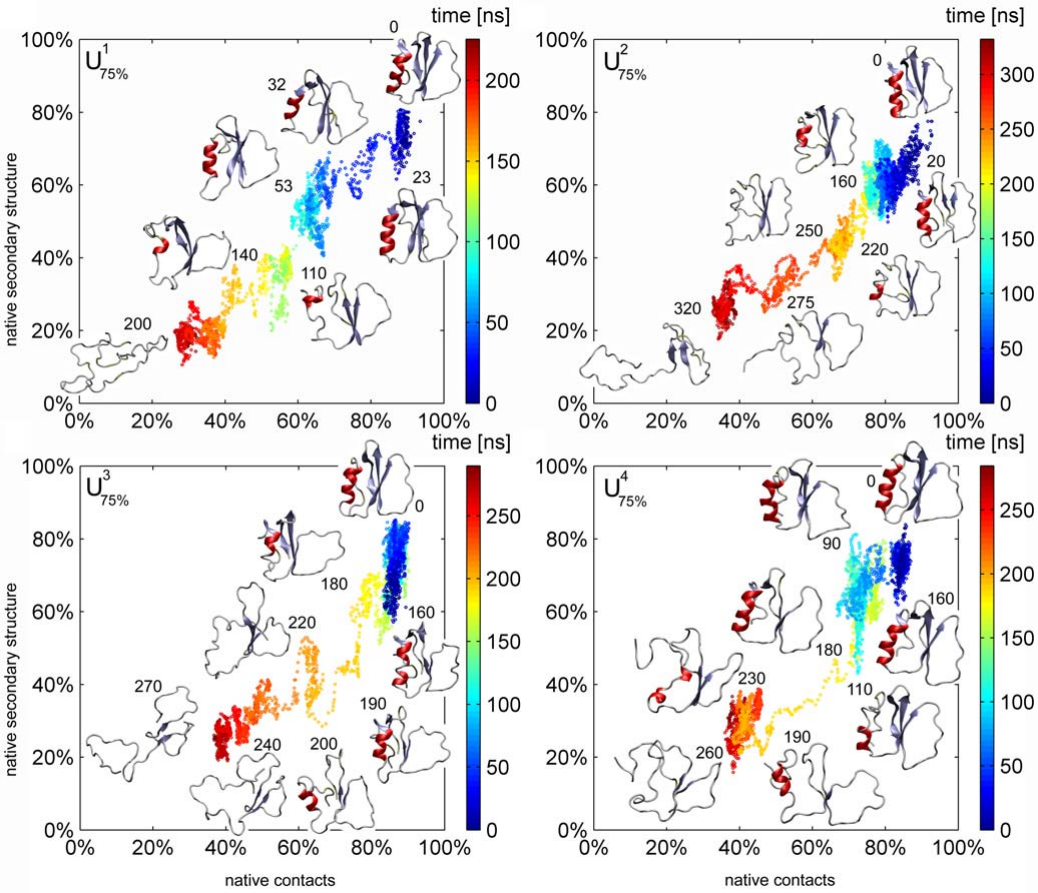
Unfolding pathways of the CI2 for the simulations in urea_75%_, displayed as native secondary structure content versus native contact content. The numbers next to the protein structures denote the respective time of the snapshot in ns.

In our simulations, the CI2 protein unfolds reproducibly in urea_75%_ (all four simulations) and urea_50%_ (all five simulations) which allows us to analyze unfolding pathways in more detail. To this aim, Figure 4 shows the unfolding pathways observed for the four simulations in urea_75%_. Here, the unfolding pathway is characterized by the fraction of native secondary structure versus fraction of native tertiary structure, measured by the fraction of native contacts. (The respective data for urea_50%_ is provided as Figure S1 in the Supporting Information). In each of the nine cases, starting from the folded state (top right), the protein undergoes conformational changes eventually leading to denaturation and unfolding in all nine trajectories.

We first describe one unfolding trajectory (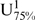) in detail, and subsequently discuss common features and differences of all nine unfolding trajectories. In simulation 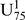, reversible fluctuations of the secondary-structure (*β*-strand 3, ILE57-ARG62) trigger the first unfolding step. After 29 ns, a part of the coil region between *β*-strand 1 and *β*-strand 2 reorients. In particular, the sidechains of THR36, ILE37 and VAL38 rotate by about 180°, which apparently triggers, at 30 ns, a subsequent flip of the turn region formed by residues 22–25. This irreversible and fast unfolding step implies significant loss of native contacts and is followed by a longer phase of 80 ns during which the *α*-helix (res. 13–22) unfolds, with the ALA-rich region (ALA14, ALA15, ALA16) unfolding last at 110 ns. Subsequently, the turn (and former *α*-) region between residues 18–25 detaches from the protein core, while the ALA-region of the helix undergoes several partial refolding and unfolding events. Between 140 ns and 150 ns, further global unfolding rearrangements of the tertiary structure occur. At 150 ns, unfolding is completed with the disruption of *β*-strands 2 (res. 46–52) and 3 (res. 56–62).

### Common Unfolding Features

Whereas the sequence and all the details of the described unfolding events are not necessarily similar in all unfolding trajectories, several common features emerge. In all simulations, unfolding proceeds stepwise, with alternating phases of loss of secondary and tertiary structure. In none of the simulations, both structure levels are seen to break down simultaneously; also not seen is the complete loss of one structure level before the other. Often, meta-stable parts of the trajectory, each sampled for a longer time (typically 100 ns and longer) and characterized by reversible fluctuations, are connected by fast transitions (about 5 ns), during which irreversible loss of native contacts occurs. Such alternating stepwise unfolding pattern is consistent with the nucleation-condensation mechanism of folding for the CI2 protein which has been derived from *φ*-value analysis [49].

In summary, a sequence of alternating unfolding steps is observed, which supports unfolding models that assume a strong coupling between tertiary and secondary loss of structure. We would like to emphasize that the sequence of meta-stable states seen in our simulations is consistent with the fact that CI2 is a two-state folder [27], because the observed transient states are both too short-lived and too heterogeneous to be resolved in current ensemble- or equilibrium-unfolding experiments.

### Onset of Unfolding

**Figure 5:**
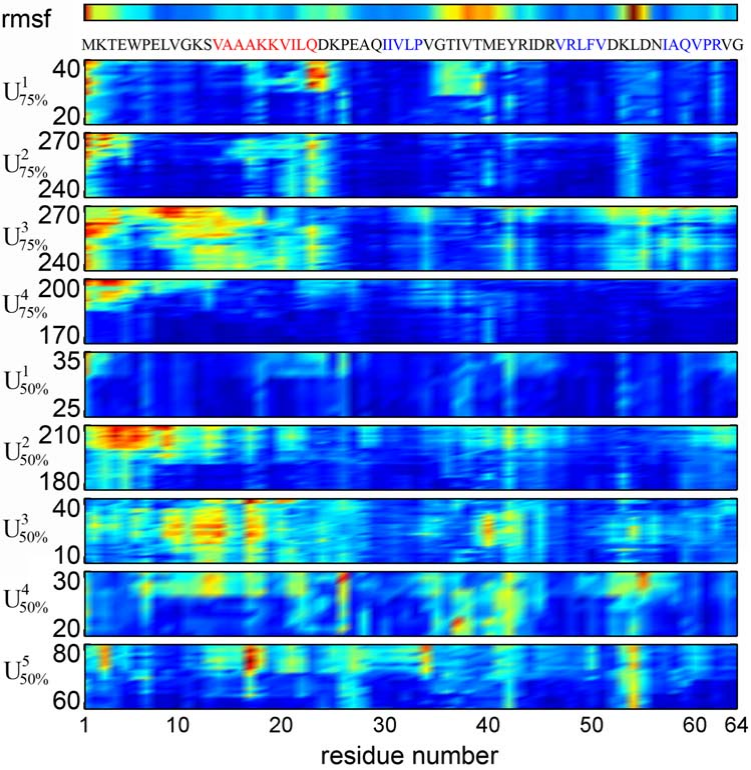
Per-residue C*_α_*-RMSD in the initial unfolding phases. Blue corresponds to low, red to high RMSD. The numbers on the left denote the start and end times of the respective displayed trajectory segment in ns. Top row: root-mean-square-fluctuations per residue in the native state. In the one-letter sequence code below, red marks the *α*-helix and blue *β*-strands.

Next, we investigated whether regions of the CI2 exist where unfolding is particularly likely to start. To this end, the RMSD per residue was calculated for the initial phase of unfolding (defined by a significant increase in the SAS from the native value) for each of the simulations U_75%_ and U_50%_ (Figure 5). For comparison, the top row shows the root-mean-square-fluctuations per residue in the native state (simulation W^300*K*^). Many initial unfolding steps are seen to occur in regions that exhibit large fluctuations already in the native state in water at 300 K. Examples are the C-terminal end of the *α*-helix (res. Q22) and the adjacent turn-region (res. D23–E26, simulations 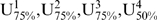), as well as the coil- and turn-regions between *β*-strands 2 and 3 (simulations 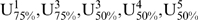). In contrast, regions that show only small fluctuation in the native state, e.g. res. 5–18 in simulations 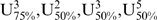, tend to unfold later. In summary, no unique unfolding “hot-spot” is found, but rather several regions where unfolding likely begins.

### Common Transient Structures

This observation led us to investigate whether common transient structures or putative intermediates exist in the unfolding pathways. To this end, for every unfolding trajectory *i*, the RMSD was calculated with respect to every structure *X_j_* (*t*) (with a time resolution of Δ*t* = 100 ps) of each of the other unfolding trajectories *j* (data not shown). In this analysis, conformations which occur in trajectory *i* as well as in trajectory *j*, would be revealed by a minimum in the respective RMSD. Unexpectedly, no pronounced minima were found, which indicates that no pair of trajectories shares common global structures, and that unfolding proceeds structurally different in all nine cases. Rather, during unfolding as well as after complete denaturation, the protein explores quite different regions of phase space. This finding further implies that the transition state ensemble consists of conformations which are structurally more heterogeneous than the thermal fluctuations of the native state. These results are consistent with the previous observations of a broad transition state ensemble [50] and the fact that the CI2 protein is a two-state folder without pronounced intermediates [27].

### Transition State Ensemble

We therefore attempted to analyze the transition state (TS) ensemble in more detail. Although the TS ensemble can not be rigorously defined from the nine trajectories at hand, a reasonable estimate can be given. To that end, we calculated the (non-equilibrium) density *ρ* of states for the SAS as reaction coordinate for each of the nine unfolding trajectories, which served to provide a rough free energy estimate, −*kT* log *ρ*. In all nine trajectories, the native state showed up as a minimum at low SAS values, with an adjacent clear maximum, which served to locate the TS (data not shown). In most simulations, this maximum was consistently seen at an increased SAS of 3–5 nm^2^. This agreement suggests that our approach provides a reliable estimate for the TS.

In all nine simulations, the overall structure of the TS is found to be similar to that of the native state (about 70% native contacts), but more expanded, in agreement with previous experimental [51]–[53] and simulation results [11],[54]. The *α*-helix is still intact, albeit with its central region bent away from the molecule's center in most simulations, whereas the *β*-sheet is already partially disrupted in most cases. In agreement with a previous simulation study [11], we find the TS ensemble to be heterogeneous with respect to the loops, turns, and terminal regions. After the TS, unfolding proceeds in six out of the nine trajectories with disruption of the *β*-structure before unfolding of the *α*-helix; conversely, in the remaining three simulations, the *α*-helix unfolds before the *β*-strands. In all cases, the time span between *α*- and *β*-disruption was rather short; therefore, no defined sequence of the two processes was established.

### Residual Structure in the Denatured State

We finally focus on the residual structure in the denatured state. In particular, we investigate a possible polyproline II helix structure (PP*_II_*, *φ* = −75°, *ψ* = 150°) which has been suggested as prevalent configuration for the denatured ensemble from CD-spectroscopy results [55]. Recently, this suggestion has gained considerable attention due to accumulating evidence for residual structure of denatured proteins [56]–[59]. We note that the sampling of the denatured state is very limited in our simulations (≈650 ns in total for urea_75%_), such that we expect this analysis to provide rough estimates rather than accurate numbers.

**Figure 6:**
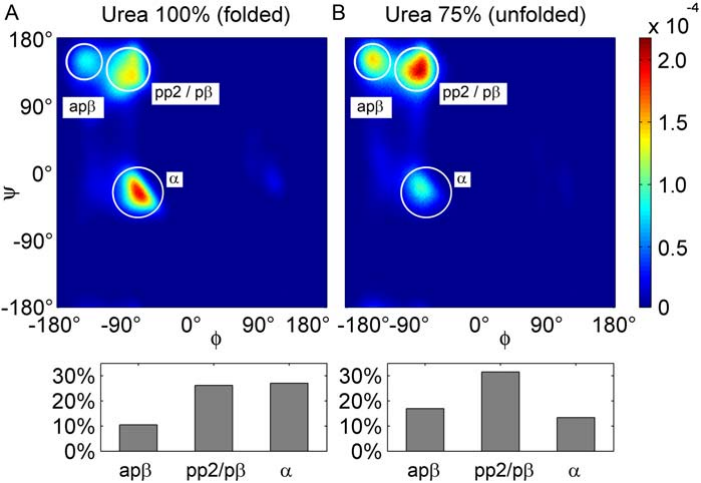
Ramachandran plots for (A) CI2 in urea_100%_ (folded state), (B) CI2 in urea_75%_ (unfolded state). The white circles show the areas which have been used for the calculation of populations densitites, which are shown in the lower panel for antiparallel *β*-sheet (“ap*β*”), PP2 or parallel *β*-sheet (“pp2/p*β*”), and helical (*α*) configurations.

Figure 6 shows the Ramachandran plot for the folded CI2 protein (averaged over all simulations with urea_100%_, panel A), and the denatured protein (averaged over the denatured ensemble in all simulations with urea_75%_, panel B). Similar distributions of the folded or unfolded ensemble were seen in the other simulations (data not shown).

As expected, the native state predominantly occupies three regions in (*φ*, *ψ*) space; the *α* region around (−70°, −27°, “*α*”), as well as the *β*-sheet regions around (−83°, 128°, parallel or PP*_II_*, “pp2/p*β*”) and (−142°, 149°, antiparallel, “ap*β*”).

For the denatured protein, the same three regions are populated, although with different occupancies (Figure 6B). In particular, the PP*_II_*/p*β* region becomes the most populated one, particularly when the denatured protein is very extended (SAS >40 nm^2^), which supports the pronounced role of this secondary structure element. However, the other two regions remain populated: the population of the antiparallel-*β* region increases from 11% to 17%, while the population of the *α*-region decreases significantly from 27% to 13%. Note that the presence of these backbone angle configurations does not imply correctly formed secondary structure elements in the denatured state.

The unambiguous classification of PP*_II_* from (*φ*,*ψ*) is complicated by the fact that other secondary structure elements share similar backbone configurations. Therefore, several different definitions to calculate PP*_II_* content have been developed and applied in the past. Integration over the pp2/p *β*-peak in Figure 6 yields an increase of 26% to 32% relative population. With the definition from Jha et al [60] (−100°<*φ*<0°, 50°<*ψ*<280°), we found a PP*_II_* population of ca. 35% in the denatured structures as compared to ca. 30% for the folded CI2. A third definition (−120°<*φ*<60°, 120°<*ψ*<240°), from Makowska et al [59], yields similar results with increase from 35% to 40% *PP_II_*. Hence, for all three definitions, we observed a pronounced, but not absolute prevalence of PP*_II_* configuration in the denatured ensemble. This finding corroborates recent results by Makowska et al. [59], who argued that PP*_II_* might be one of several possible backbone conformations in the denatured state.

## Discussion

To elucidate whether polar or, in contrast, apolar urea-protein interactions are the key driving force for urea-induced denaturation, thought experiment simulations were performed, in which the respective denaturation strengths of hyperpolar urea (with strengthened polar interactions) or hypopolar urea (with strengthened apolar interactions) were compared. To this end, the CI2 protein was simulated in water, in regular urea, and in hypo- and hyperpolar urea, which was realized by scaling the partial charges of the urea force field.

In all nine simulations with reduced urea polarity, the CI2 protein unfolded within 300 ns. In contrast, the protein remained stable in the simulations with increased urea polarity, and the folded state was found to be even slightly more compact than in water. These results provide strong evidence that interactions with less polar parts—rather than polar interactions—are the main driving force for urea-induced protein denaturation. Together with previous results [46], a coherent picture for urea-induced protein denaturation emerges. Urea molecules accumulate around less polar side chains and exposed backbone, forming an interface between less polar protein surface and water. The resulting displacement of water molecules from the protein surface into bulk water is entropically and enthalpically favorable and reduces the hydrophobic effect, such that unfolding of the protein becomes favorable. The ability of urea to form hydrogen bonds to the protein backbone is not the main driving force for denaturation, but contributes to the overall energetics by preventing unsatisfied hydrogen bond sites at the protein backbone. This view is also in agreement with recent spectroscopic results which provide evidence against the dominant role of polar interactions and hydrogen bonds [61].

It is interesting to note a relation to the mechanism of chaperone-mediated folding. Recent investigations of the chaperone GroEL [62] provide support for the suggestion that the hydrophobic environment of the open state of GroEL facilitates unfolding, whereas the hydrophilic environment of the closed state of GroEL facilitates folding [63],[64]. In our simulations, we also find a more hydrophobic environment (aqueous solution of hypopolar urea) to facilitate unfolding, and a more hydrophilic environment (aqueous solution of hyperpolar urea) to facilitate folding.

For regular urea, the preferences of the 20 natural amino acids for contacts with either urea or water were largely similar to those found previously for tripeptides [46]. In particular, less polar residues interacted preferentially with urea, whereas polar and particularly charged residues had stronger preferences for interaction with water. As expected, the characteristics of this interaction profile were amplified for hypopolar urea, and inverted for hyperpolar urea.

The observation that the CI2 protein does not unfold within several hundred microseconds in urea with regular charges is consistent with the measured millisecond unfolding time [47]. We could not reproduce the complete nanosecond-unfolding seen in previous simulations [11], which however employed a cutoff-approximation for the long-range electrostatics.

On the structure level, our simulations suggest that denaturation proceeds rather heterogeneously and not via narrow, distinct pathways. In particular, unfolding of the CI2 was observed to start in stochastically one of several regions rather than one. However, regions with large structural fluctuations already in the folded state often turned out to be primary unfolding regions. Moreover, the nine unfolding pathways in the simulations with urea_75%_ and urea_50%_ turned out to share no common conformations during unfolding, which is consistent with the fact that CI2 is a two-state-folder without meta-stable folding intermediates. This heterogeneity of unfolding pathways prompts us to suggest an “inverted funnel”-scenario for the unfolding energy landscape, with multiple pathways leading from the narrow mesa of the folded state down to the relatively flat and extended region of the denatured ensemble.

Whereas no shared conformations were found in the different unfolding pathways on the detailed level, more general common features of the unfolding process emerge. In particular, in most of the simulations unfolding was observed to proceed with alternating and sequential loss of secondary and tertiary structure. This finding is consistent with the coupling between secondary and tertiary structure formation in the nucleation-condensation folding process of the CI2 inferred from spectroscopic and mutation studies [49],[65],[66]. It further suggests that the processes of structure-formation during folding and structure-loss during denaturation share common features.

Finally, our simulations allowed us to analyze the residual structure in the denatured state. Overall, relatively little residual secondary structure was seen, in agreement with previous CD studies [65]. Polyproline II turned out to be the most prominent, however not dominant residual structure in the unfolded ensemble. This finding supports the recent suggestion that polyproline II is one of several possible backbone conformations in the denatured state [59]. *α*-helical structure was found to be drastically reduced, whereas the population of *β*-sheet like backbone conformations was even slightly enhanced in the denatured state. Should such increase of *β*-sheet like backbone conformations turn out to be a common feature of unfolded protein ensembles, it might be relevant for the structural understanding of *β*-amyloid formation.

## Supporting Information

Figure S1 CI2 unfolding pathways in urea with 50% partial charge scaling.
(2.80 MB PDF)

Text S1 Extent of self-diffusion slowdown and urea aggregation.
(2.80 MB PDF)
