# Supplementary material for: Polar or Apolar—The Role of Polarity for Urea-Induced Protein Denaturation
Source: PLoS Comput Biol. 2008 Nov 14;4(11):e1000221. doi: 10.1371/journal.pcbi.1000221 (PMC2570617; doi:10.1371/journal.pcbi.1000221)
Supplement: Figure S1 — CI2 unfolding pathways in urea with 50% partial charge scaling. (2.80 MB PDF) [file pcbi.1000221.s001.pdf]

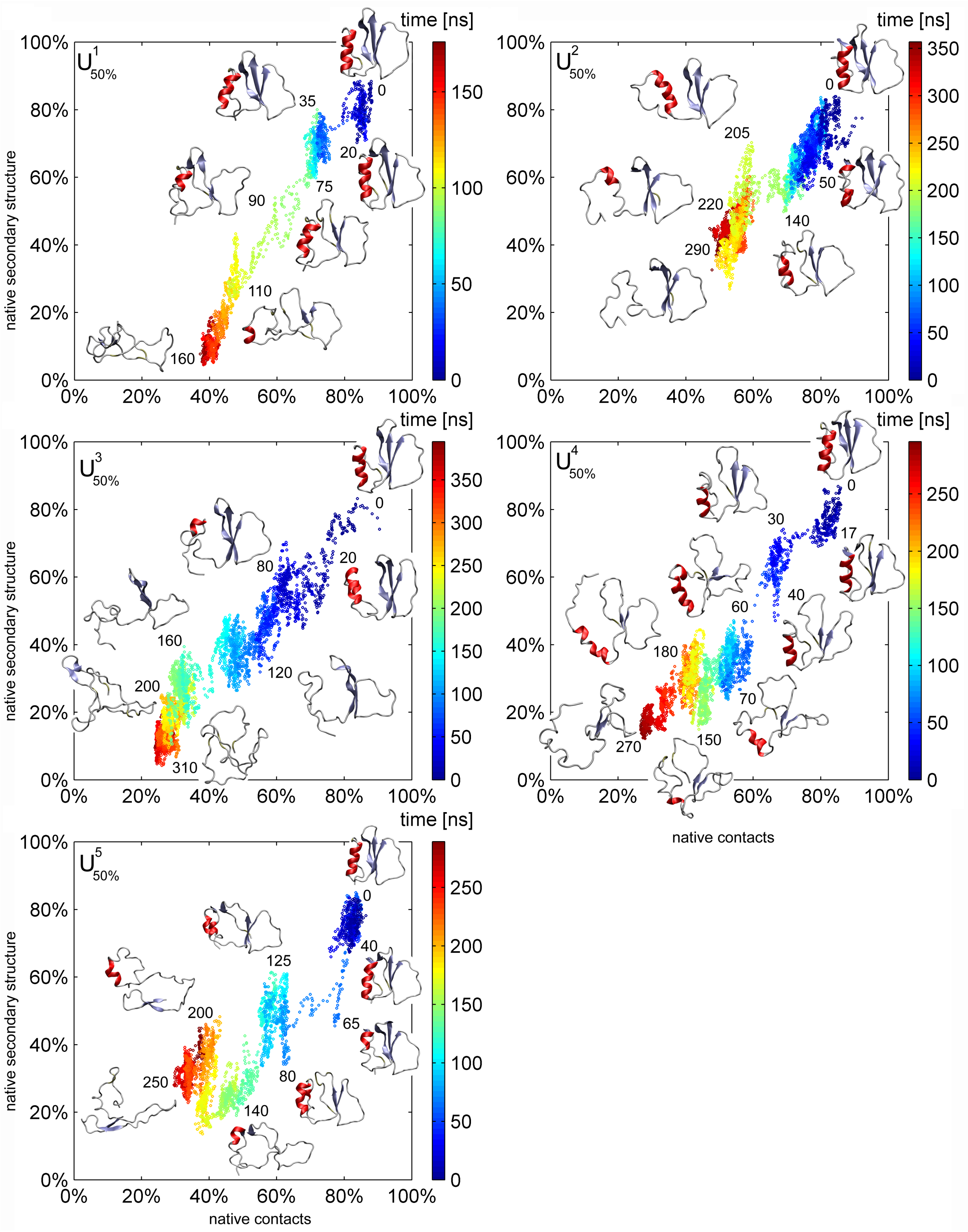

FIGURE S2. Unfolding pathways of the CI2 for the simulations in urea$_{50\%}$, displayed as native secondary structure content versus native contact content. The numbers next to the protein structures denote the respective time of the snapshot in ns.
