# Supplementary material for: Polar or Apolar—The Role of Polarity for Urea-Induced Protein Denaturation
Source: PLoS Comput Biol. 2008 Nov 14;4(11):e1000221. doi: 10.1371/journal.pcbi.1000221 (PMC2570617; doi:10.1371/journal.pcbi.1000221)
Supplement: Text S1 — Extent of self-diffusion slowdown and urea aggregation. (2.80 MB PDF) [file pcbi.1000221.s002.pdf]

Aggregation was quantified for hypopolar urea by calculating the urea *distribution entropy*,

$$S_{dist} = -\int dV \rho(x, y, z) \log \rho(x, y, z) \quad ,$$

where V is the box volume, and ρ(x,y,z) the local urea density after kernel density estimation. The width of the gaussian kernel function was chosen such that the full-width-half-maximum was equal to the average distance of N equidistantly distributed particles in the volume V, where N was given by the number of urea molecules. The distribution heterogeneity H_{dist} for a distribution X was then calculated by

$$H_{dist}(X) = \frac{S_{dist}(X) - S_{dist}^{max}}{S_{dist}^{min} - S_{dist}^{max}} \quad ,$$

where S_{dist}^{max} and S_{dist}^{min} are the maximum distribution entropy (given by a completely homogeneous distribution of particles) and the minimum distribution entropy (given by a δ-distribution of particles), respectively.

To investigate possible artifacts from self-diffusion slowdown for hyperpolar urea, the urea self-diffusion coefficient was calculated via the mean-square-displacement D of urea molecules.

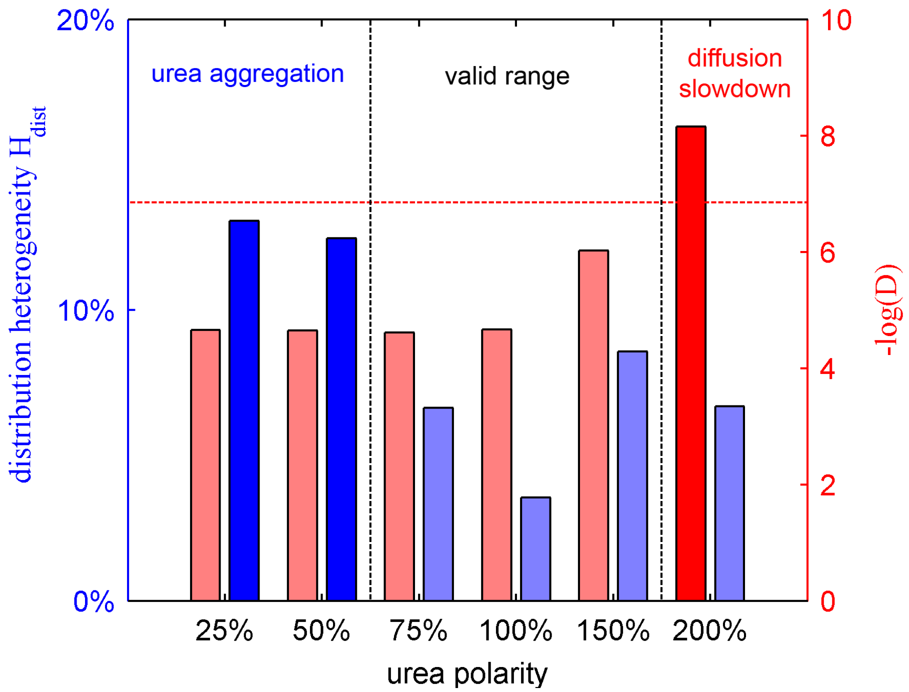

FIGURE S1. Side-effects of urea partial charge scaling. At 200 % partial charge scaling, urea self-diffusion (red bars) is more than an order of magnitude too slow to cross the box-length within the simulation time (indicated by the red dashed line). Scaling of the partial charge to 25 % and 50 % induces a significant degree of urea aggregation, as indicated by the urea distribution heterogeneity (blue bars).
